# A Novel Three-dimensional Mapping Device to Guide Lead Placement for Left Bundle Branch Area Pacing

**DOI:** 10.19102/icrm.2025.16104

**Published:** 2025-10-15

**Authors:** Mahmoud Ali, Lynn Erickson, Sisir Siddamsetti, Tarek Ajam, Mohammed Djelmami-Hani, Imran K. Niazi

**Affiliations:** 1 Aurora Cardiovascular and Thoracic Services, Aurora Sinai/Aurora St. Luke’s Medical Centers, Aurora Health Care, Milwaukee, WI, USA; 2 Division of Cardiovascular Medicine, University of Wisconsin School of Medicine and Public Health, Milwaukee, WI, USA; 3 Academic Affairs, Cardiovascular Research, Aurora Sinai/Aurora St. Luke’s Medical Center, Advocate Aurora Health, Milwaukee, WI, USA

**Keywords:** Three-dimensional mapping, lead placement, left bundle branch area

## Abstract

Navik 3D (APN Health, Waukesha, WI, USA) is a navigation software program that uses two-dimensional (2D) fluoroscopy images to provide three-dimensional (3D) information. Left bundle branch area (LBBA) pacing (LBBAP) is a novel physiologic pacing technique where the lead is placed in the right ventricular (RV) basal septum to capture the left bundle branch (LBB). Precise lead placement in this region can be challenging using 2D fluoroscopy. We studied the feasibility of using Navik 3D to identify the location, plane, and depth of the lead in the septum to assist with LBBAP procedures. This observational, prospective single-center study included 14 patients undergoing LBBAP. Navik 3D was used to identify the LBBA, RV septum, RV apex, and lead position in three dimensions using two orthogonal 2D views. The 3D images were overlaid on real-time, gated fluoroscopic images for navigation of the lead. Images of the 3D locations and successful or unsuccessful lead locations were projected onto 2D fluoroscopic images, allowing for repositioning if necessary. All attempted patients had successful LBBA lead implants. An LBB potential was recorded in 61.5% of the patients. Selective LBBAP was achieved in 85% of the patients. The mean QRS duration postimplant was 129.8 ± 13.1 ms. The mean left ventricular activation time (stimulus R-wave peak in V6) postimplant was 75 ± 12 ms. No acute complications were recorded. 3D localization of the LBBA using the Navik 3D mapping system was feasible and may assist with more appropriate LBBA lead placement.

## Introduction

Left bundle branch (LBB) area (LBBA) pacing (LBBAP) is a novel physiological pacing technique that attempts to normalize electrical activation of the ventricles.^1–4^ It has emerged as an alternative to biventricular pacing in patients with an indication for cardiac resynchronization therapy.^5^ Successful LBBAP is achieved by positioning the ventricular lead near the LBB on the right side of the interventricular septum and advancing the lead through the septum to reach the left bundle. Precise lead placement in this region can be challenging owing to individual anatomical variations. Fluoroscopy is traditionally used to guide lead placement, but it provides limited two-dimensional (2D) information, which may lead to prolonged procedure and fluoroscopy times and less successful implants.

The Navik 3D navigation system (APN Health, Waukesha, WI, USA) is an approved software program that allows for real-time display of cardiac maps in various formats, including anatomical, electrical activation, and voltage maps. Navik 3D was first described in a canine model and further tested as a means to locate the cryoballoon and esophageal temperature probe in three-dimensional (3D) images.^6,7^ In short, the system receives image data from a standard electrophysiology laboratory 2D fluoroscopic X-ray system along with an electrocardiogram (ECG), electrogram (EGM), and other data. Standard 2D fluoroscopy can assess and locate radiopaque objects by using the X (left-right) and Y (up-down) coordinates, but the Z (back-front) coordinate, which completes the 3D aspect, is unknown. Navik 3D software uses the idea that a fixed-size, radiopaque object projects a shadow, and if the distance between the source and the detector is known, as it is in standard electrophysiology (EP) labs, the Navik 3D system can use this information to calculate the position of the object in the *Z* axis, and thus calculate the 3D (X, Y, and Z) location of the object. This allows the user to create 3D images of objects in the heart from the standard 2D fluoroscopy, without relying on special catheters or mapping technologists to create the map. The idea is to create a cost-effective and user-friendly approach to localizing catheters and other EP tools of known size that are radiopaque in 3D. By design, the system is a cost-effective alternative because of its reliance on the software that does not need any special catheters or patches to localize a device in three dimensions using 2D fluoroscopy.

**Figure 1:**
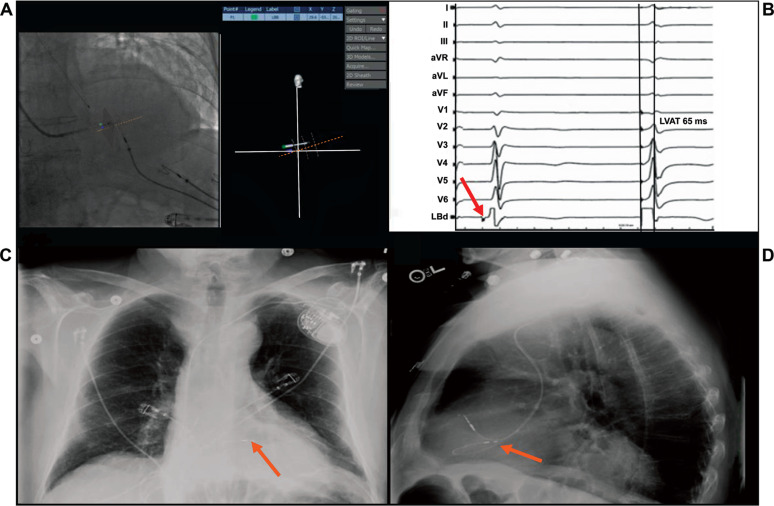
Use of a three-dimensional mapping device to guide left bundle branch area pacemaker implant. **A:** Fluoroscopy image on Navik 3D showing the left bundle branch pacing area on the septum and right lead trajectory (orange dashes). **B:** Electrogram showing the left bundle potential at lead location (red arrow) and paced beat with left ventricular activation time of 65 ms. **C and D:** Two-view chest X-ray postimplant showing lead location (orange arrow).

APN Health has also developed investigational software that may augment 2D fluoroscopy in pacing lead placement for LBBAP **(Figure 1)**. This development could assist with identifying the correct angle at which the lead enters the interventricular septum and also determine the depth of lead penetration into the septum.

In this study, we aimed to describe the feasibility of using an investigational version of the Navik 3D software to navigate LBBA lead placement. Standard-of-care single-plane fluoroscopy was used in parallel with this to achieve LBBA lead placement.

## Methods

### Study design

This prospective, observational, single-center study included patients ≥18 years of age undergoing LBBA pacemaker implantation. Subjects were enrolled between September 2022 and May 2023. Patients received LBBA pacemaker implantation using standard-of-care 2D fluoroscopy in parallel with the Navik 3D investigational software. The research reported in this paper adhered to the Declaration of Helsinki as revised in 2013, and all patients provided informed consent. This project was approved by the WCG (WIRB-Copernicus Group) Institutional Review Board.

### Left bundle branch area pacing

**Figure 2:**
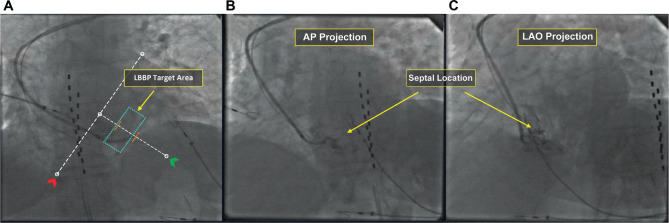
Identifying the location of interest in the left bundle branch area. **A:** Right anterior oblique projection showing the tricuspid valve line (red arrowhead), right ventricular apex line (green arrowhead), and left bundle branch pacing target area located between the proximal and the middle third of the septum (blue rectangle). **B and C:** Septal location with contrast injection in anteroposterior and left anterior oblique views.

**Figure 3:**
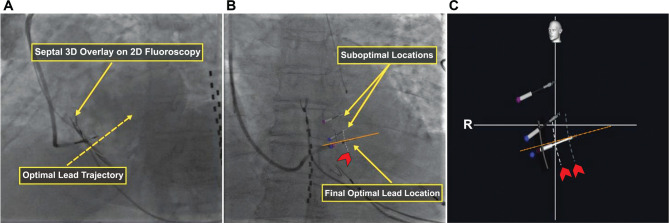
Reconstruction of the septum in three dimensions (3D) overlaid in fluoroscopy. **A:** The 3D reconstruction of the septum overlay on two-dimensional (2D) fluoroscopy, which is generated from two views of 2D fluoroscopy images **(Figures 2B and 2C)**. The system draws the optimal lead trajectory perpendicular to the septum (dashed orange arrow). **B and C:** Two suboptimal locations with repositioning to the optimal lead location. The depth of the lead markers on the septum is represented with dashed lines (red arrowheads); each dashed line represents 6 mm in depth.

The LBBAP procedures were performed under local anesthesia and intravenous sedation. After obtaining access through the left axillary/subclavian veins, a guidewire was introduced through the sheath and placed with the tip in the right ventricular (RV) apex using 2D fluoroscopy in the right anterior oblique (RAO) projection to mark the RV apex. The Navik 3D system marked three strategic points (the border of the right atrium, the meeting point of the left ventricle [LV] and the pulmonary artery, and the RV apex) to draw a tricuspid valve line connected to the RV apex **(Figure 2A)**. The area of interest for the LBBA was then identified and represented by a marked rectangle projected into live 2D fluoroscopy in RAO projection **(Figure 2A)**. A C304 or C315 His delivery sheath (Medtronic, Inc., Minneapolis, MN, USA) was then advanced over the wire to the area of interest. Contrast was injected through the sheath, and images were obtained in anteroposterior and left anterior oblique views, separated by at least 20°. The acquisition time for each image was 3 s to allow for gating **(Figures 2B and 2C)**. This mapping method allowed the Navik 3D system to create a construct of the interventricular septum localization and then project it on the live 2D fluoroscopy images **(Figure 3A)**. Serial plotting and refinement were performed to create the optimal lead trajectory on the septum and mark the depth of the lead **(Figure 3A)**.

Similarly, the tip of the sheath and the radiopaque markers on the lead were also marked **(Figure 3A)**. This step allowed real-time visualization of the lead position and orientation in relation to target anatomy. If repositioning was required, the initial unsuccessful position was marked. A new target site could then be identified, accounting for the prior location **(Figures 3B and 3C)**. Lead depth markers are drawn by the system as dashed lines on the fluoroscopy image. Each dashed line represents a 6-mm depth from the septum **(Figures 3B and 3C)**. A model 3830 lead (Medtronic, Inc.) was then advanced through the sheath, positioned at this site, and directed to the LBBA previously marked on the Navik 3D system. The septal target site was identified, where the paced QRS morphology demonstrated a W pattern in lead V1. The lead was then screwed into the interventricular septum using rapid clockwise rotations. Unipolar pacing was conducted during fixation to monitor the paced QRS morphology and pacing impedance. The LV activation time (LVAT) and intrinsic and paced QRS durations for LBBAP were measured. Lead parameters were then tested in unipolar and bipolar configurations. The sheath was then slit, and the slack was adjusted under fluoroscopy.

### Confirmation of left bundle branch area capture

Confirmation of LBBA capture was completed using ECG and EGM criteria **(Table 1)**.^8,9^

**Table 1:** Left Bundle Branch Area Capture Electrocardiography Confirmation Criteria

Paced QRS morphology with RBBB morphology: QR or rSR’ Presence of LBB potential Pacing stim-LVAT Stimulus to the peak of the R-wave in V5 or V6 ≤75 ms in normal QRS Abrupt shortening by >10 ms with LB capture Short and constant at high (5 V) and low (1 V) outputs Determination of S- and NS-LBBAP Selective LBBAP: Stim-QRS latency seen. Discrete local EGM separate from stimulus artifact seen NS-LBBAP: No stim-QRS latency. No discrete local EGM separate from stimulus artifact Evidence for direct LBB capture Stim to retrograde His time from unipolar tip versus ring pacing Retrograde VA time from unipolar tip versus ring pacing

*Abbreviations:* EGM, electrogram; LBB, left bundle branch; LBBAP, left bundle branch area pacing; LVAT, left ventricular activation time; NS, non-selective; RBBB, right bundle branch block; S, selective.

The ECG and EGM data were analyzed. ECGs before and after pacing were evaluated. The LVAT was measured from the stimulus artifact on the surface 12-lead ECG to the peak of the R-wave in lead V6.

### Statistics

Data were summarized using the mean (±SD) for continuous variables and n (%) for categorical variables.

## Results

The study initially included 14 subjects undergoing LBBA pacemaker implantation. Subjects presented with either a complete heart block (n = 6) or sinus node dysfunction (n = 8). The procedure was abandoned in 1 of the 14 patients owing to a severely enlarged RV size and the inability to reach the intraventricular septum using both the pre-shaped and adjustable curve sheaths. Patient characteristics for the attempted patients (n = 13) are depicted in **Table 2**.

**Table 2:** Baseline Characteristics

Patient Characteristics	All Patients (n = 13)
Age (years)	76.1 ± 8.8
Female sex	5 (3.8)
Caucasian	12 (92.3)
African American	1 (7.7)
Smoking	5 (38.5)
Hypertension	11 (84.6)
Diabetes	1 (7.7)
Hyperlipidemia	11 (84.6)
Chronic kidney disease	4 (30.8)
Stroke/TIA	2 (15.4)
Atrial fibrillation	8 (61.5)
Pulmonary hypertension (>moderate)	8 (61.5)
LV ejection fraction (%)	56.4 ± 9.9
LV end-diastolic diameter (cm)	4.9 ± 0.60
Intraventricular septal diastolic thickness (cm)	1.15 ± 0.17
Dilated RV	3 (23.1)
Mitral regurgitation (>moderate)	0 (0)
Tricuspid regurgitation (>moderate)	1 (7.7)
Dilated LA	8 (61.5)
Baseline QRS (ms)	97.8 ± 21.9

*Abbreviations:* LA, left atrium; LV, left ventricular; RV, right ventricular; TIA, transient ischemic attack. Continuous variables are expressed as mean ± standard deviation values, and categorical variables are expressed as n (%). Dilated LA size, volume index > 27 mL/m^2^; dilated RV size was defined as an RV base length of >41 mm and an RV mid-level base length of >35 mm in end-diastole.

**Figure 4:**
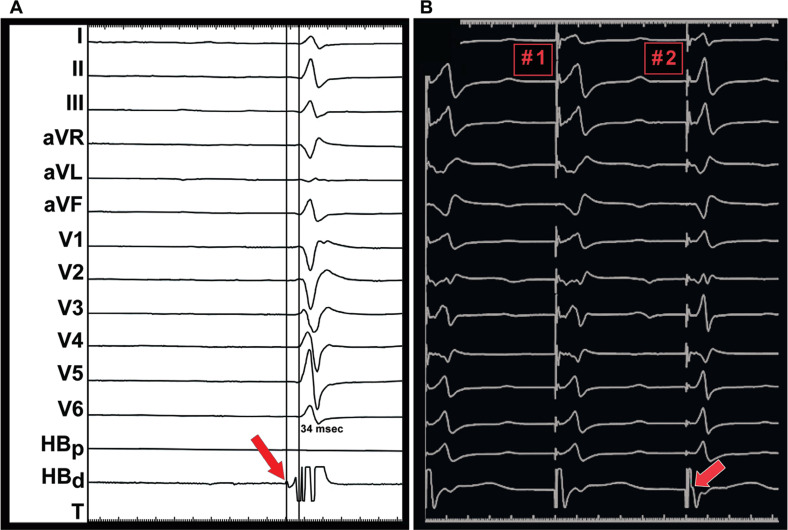
Demonstration of the left bundle potential and left bundle pacing. **A:** Conducted beat with lead at the left bundle location showing left bundle potential (red arrow). **B:** Beat #1 shows non-selective left bundle pacing. Beat #2 shows selective left bundle pacing.

LBBA lead placement was successful in all subjects. Two patients had high thresholds initially, and the lead was repositioned in both patients under Navik 3D guidance with subsequent improvement. At the end, all patients had acceptable pacing thresholds of <2 V at 0.4 ms. The LBB potential was recorded in eight (61.5%) patients **(Figure 4A)**. Selective LBBAP was achieved in 11 (84.6%) patients **(Figure 4B)**. The mean LVAT was 75.6 ± 12.8 ms, and the mean post-QRS duration was 129.8 ± 13.1 ms.

Seven patients underwent LBBAP alone, and the remaining six patients also underwent concomitant atrioventricular junction ablation. In the seven patients undergoing sole LBBAP, the mean fluoroscopy time was 16 ± 8.4 min, and the procedure time was 68 ± 35.9 min.

At the 1-month follow-up, all leads were functioning, with no reported incident of lead dislodgement. There were no significant procedure-related complications. **Table 2** shows patient characteristics.

## Discussion

The LBBAP is a new physiologic pacing method; equipment and implant techniques are in evolution. High success rates are reported by the most experienced implanters,^2,8,10^ but new implanters face many challenges. There are no large randomized multicenter trials that provide actual success rates in clinical practice. Using 2D fluoroscopy alone to guide LBBA lead positioning in this anatomically complex region has many limitations. Fluoroscopy alone only provides 2D information in a fixed projection; meanwhile, the actual 3D relationships between the lead and septal structures are difficult to appreciate. The most experienced implanters can intuit the lead–septum relationship from 2D fluoroscopy based on long experience, but these skills are not easily or rapidly acquired. The Navik 3D integration addresses these challenges by reconstructing enhanced visualization of the lead trajectory and target region, which can improve placement accuracy by ensuring perpendicular placement of the lead in the septum. This can be done without specialized catheters or industry representatives but with standard 2D fluoroscopy and EP lab staff. The cost of this software package is a fraction of more sophisticated 3D mapping systems based on impedance measurements or perturbations in the magnetic field, and there are no recurring costs with each case.

The Navik 3D system also helps directly determine the depth of the lead tip within the septum as it is progressively approaching the LBBB. This guidance may improve outcomes and may prevent complications related to the procedure, such as septal perforation and lead dislodgements (see **Figures 3B and 3C**).^10,11^ Navik 3D might also help reposition the lead when the initial attempt was unsuccessful by marking the location of the unsuccessful site. Marking the location of the unsuccessful site using Navik 3D may prevent repeat trauma to the same suboptimal sites and reduce procedure and fluoroscopy times.

This study demonstrates that Navik 3D mapping and navigation may be effectively incorporated into LBBAP procedures. Our results validate the feasibility of using Navik 3D navigation for these procedures. We consistently achieved ECG patterns indicating LBB capture, with short-paced LVAT confirming appropriate lead positioning near the native conduction system.

There are several limitations to this single-center observational study. The sample size was small, and the long-term clinical outcomes were not evaluated. Comparative studies using a control group would have been valuable to determine if Navik 3D navigation improves LBBA success rates, reduces radiation exposure and procedure time, or provides other objective benefits over traditional fluoroscopic guidance. A cost comparison is also lacking in this study but would be beneficial in future studies. Using the system itself involves a learning curve, which could affect initial results.

## Conclusions

The Navik 3D mapping and navigation allow localization of the RV septum and lead trajectory during LBBAP procedures. Our initial experience indicates this platform may aid in precise lead positioning and relocation when required. This approach may be useful for optimizing the implantation of LBBAP systems. Further studies are warranted using a control group to fully define its utility and impact on outcomes.
